# Supplementary material for: Loss of CARM1 alters the developmental programming of Glioma stem-like cells and creates a druggable NGFR/NTRK dependency
Source: bioRxiv. 2025 Apr 17:2025.04.11.647869. Preprint. [Version 1] doi: 10.1101/2025.04.11.647869 (PMC12047859; doi:10.1101/2025.04.11.647869)
Supplement: Supplement 1 [file media-1.pdf]

Supplementary Figures

Loss of CARM1 alters the developmental programming of Glioma stem-like cells and creates a druggable NGFR/NTRK dependency

Authors: Dejauwne L. Young[1,2], Jennifer Aguilan[1], Ronald Cutler[1], Stephanie Stransky[1], Joseph D. DeAngelo[1], Jacob S. Roth[1], Beata Malachowska[2], Justin Vercellino[2], Brett I. Bell[2], David Shechter[1], Philip J. Tofilon[3], Richard E. Phillips[4], Chandan Guha[2]*, and Simone Sidoli[1]*

[1]Department of Biochemistry, Albert Einstein College of Medicine, Bronx, NY 10461, USA

[2]Department of Radiation Oncology, Albert Einstein College of Medicine, Bronx, NY 10461, USA

[3]Radiation Oncology Branch, National Cancer Institute, Bethesda, MD 20892, USA

[4]Department of Neurology, The Perelman School of Medicine, University of Pennsylvania, Philadelphia, PA 19104, USA

<u>Corresponding authors:</u>

Simone Sidoli, simone.sidoli@einsteinmed.edu

Chandan Guha, cguha@montefiore.org

A.

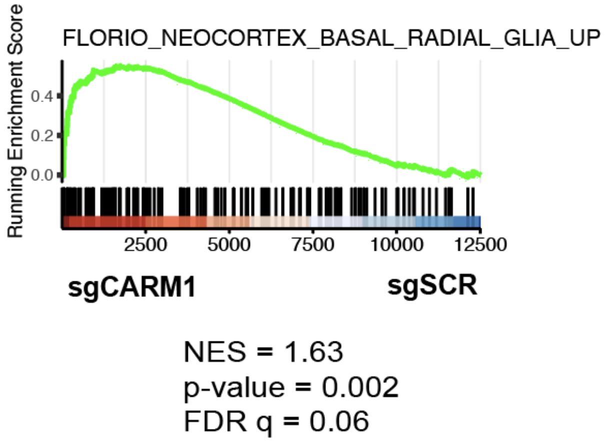

B.

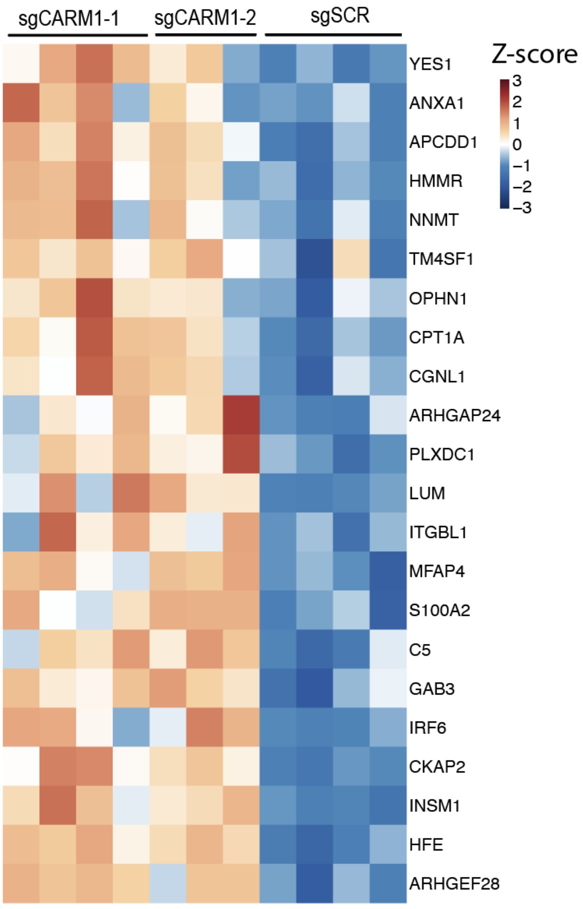

Supplementary Figure 1. A) GSEA of RNA-seq and B) heatmap of differentially expressed genes found enriched in radial glial cells from panel A.

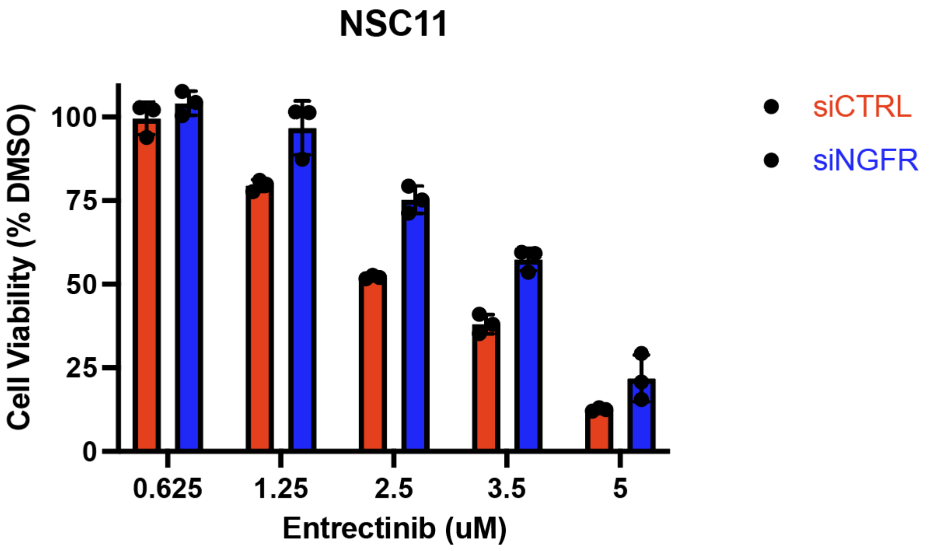

Supplementary Figure 2. Bar graphs after 4-day treatment of Entrectinib on siCTRL or siNGFR NSC11 GSCs, n = 3, data shows mean +/- SD.

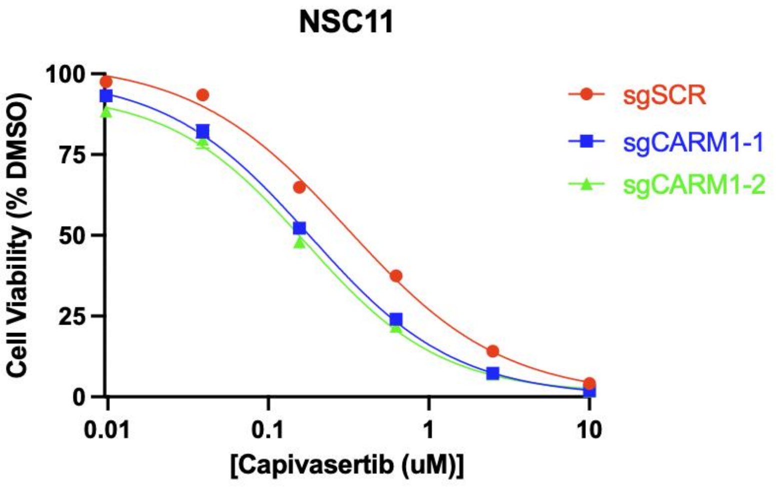

Supplementary Figure 3. Dose response curve of NSC11 sgSCR or sgCARM1 cells treated with the pan-AKT inhibitor, Capivasertib, n = 3. Data shows mean +/- SEM. **sgSCR ic50 = 312nM sgCARM1-1 ic50 = 182nM sgCARM1-2 ic50 = 171nM**
